# Oridonin Targets Multiple Drug-Resistant Tumor Cells as Determined by *in Silico* and *in Vitro* Analyses

**DOI:** 10.3389/fphar.2018.00355

**Published:** 2018-04-16

**Authors:** Onat Kadioglu, Mohamed Saeed, Victor Kuete, Henry J. Greten, Thomas Efferth

**Affiliations:** ^1^Department of Pharmaceutical Biology, Institute of Pharmacy and Biochemistry, Johannes Gutenberg University Mainz, Mainz, Germany; ^2^Abel Salazar Institute of Biomedical Sciences, University of Porto, Porto, Portugal; ^3^Heidelberg School of Chinese Medicine, Heidelberg, Germany

**Keywords:** cluster analysis, drug resistance, microarray, molecular docking, molecular dynamics, natural compound

## Abstract

Drug resistance is one of the main reasons of chemotherapy failure. Therefore, overcoming drug resistance is an invaluable approach to identify novel anticancer drugs that have the potential to bypass or overcome resistance to established drugs and to substantially increase life span of cancer patients for effective chemotherapy. Oridonin is a cytotoxic diterpenoid isolated from *Rabdosia rubescens* with *in vivo* anticancer activity. In the present study, we evaluated the cytotoxicity of oridonin toward a panel of drug-resistant cancer cells overexpressing ABCB1, ABCG2, or ΔEGFR or with a knockout deletion of TP53. Interestingly, oridonin revealed lower degree of resistance than the control drug, doxorubicin. Molecular docking analyses pointed out that oridonin can interact with Akt/EGFR pathway proteins with comparable binding energies and similar docking poses as the known inhibitors. Molecular dynamics results validated the stable conformation of oridonin docking pose on Akt kinase domain. Western blot experiments clearly revealed dose-dependent downregulation of Akt and STAT3. Pharmacogenomics analyses pointed to a mRNA signature that predicted sensitivity and resistance to oridonin. In conclusion, oridonin bypasses major drug resistance mechanisms and targets Akt pathway and might be effective toward drug refractory tumors. The identification of oridonin-specific gene expressions may be useful for the development of personalized treatment approaches.

## Introduction

Chemotherapy is a mainstay of cancer treatment in addition to surgery, radiotherapy, and antibody-based immunotherapy. Conventional chemotherapy fails for many cancer patients due to various factors, drug resistance being one of the main reason together with severe side effects. Therefore, drug research constantly attempts to improve treatment results by the preclinical development of new drugs and the optimization of therapy regimens in the clinic.

Natural products always played an important role in cancer pharmacology (Newman and Cragg, 2007), they are not only well-established cytotoxic anticancer drugs (e.g., anthracyclines, *Vinca* alkaloids, taxanes, camptothecins, etc.), but also valuable lead compounds for the development of novel targeted chemotherapy approaches (Walkinshaw and Yang, 2008; Gallorini et al., 2012; Garcia-Carbonero et al., 2013). Natural products can exert synergistic interaction with other natural or synthetic drugs (Efferth, 2017; Nankar et al., 2017; Nankar and Doble, 2017; Wagner and Efferth, 2017; Zacchino et al., 2017a,b), they can overcome drug resistance (Guo et al., 2016; Reis et al., 2016; Teng et al., 2016; Zuo et al., 2016), reduce side effects of chemotherapy and stimulate the immune system (Lacaille-Dubois and Wagner, 2017; Schad et al., 2017).

Abnormal activation of signal transduction pathways may lead to carcinogenesis, invasion, and metastasis of tumors (Leber and Efferth, 2009; Spano et al., 2012). Signaling pathways related to the epidermal growth factor receptor (EGFR) such as EGFR/PI3K/AKT-mTOR pathway command a unique position in cancer biology (Efferth, 2012). Targeting those proteins led to the development of cancer therapeutics such as erlotinib and gefitinib (EGFR inhibitors), LY294002 (PI3K inhibitor), peritosine (Akt inhibitor), rapamycin and sirolimus (mTOR inhibitors), and many others.

Amplification of the EGFR gene (with a frequency of ∼50% in glioblastoma multiforme-GBM) (Furnari et al., 2007) is often associated with a tumor-specific mutation encoding a truncated form of the receptor, which lacks the extracellular binding domain, known as ΔEGFR (also named de2-7EGFR or EGFRvIII) leading to ligand-independent, constitutive tyrosine kinase activity. Expression of ΔEGFR is connected with glioma cell migration, tumor growth, invasion, survival, and resistance to treatment, and correlates with decreased overall survival in GBM patients (Heimberger et al., 2005; Liu et al., 2010). Drug resistance mediated by EGFR is not restricted to established anticancer drugs but also occurs toward other cytotoxic compounds of natural origin. Hence, EGFR-mediated resistance may represent a general type of cellular defense mechanisms toward a broad range of toxic xenobiotics (Kadioglu et al., 2015).

A well-known tumor suppressor gene, TP53 is one of the main guardian of normal cell proliferation by preventing cells with DNA damage to proliferate. Mutations or deletions in the TP53 gene are observed in approximately 50% of human cancers, leading to impaired tumor suppressor function (Wang et al., 2017). Proliferation of cells with DNA damage rises the risk of transferring mutations to the next generation upon loss of p53 functionality; therefore, deregulation of p53 often leads to tumor formation (Khoury and Domling, 2012). Abnormal p53 status is also linked with drug resistance and chemotherapy failure (Muller and Vousden, 2013).

ATP-binding cassette (ABC) transporters play crucial role to regulate absorption, distribution, metabolism, and excretion in normal tissues (Natarajan et al., 2012). Overexpression of certain ABC transporters such as ABCG2/BCRP and ABCB1/Pgp in tumor cells is linked with resistance to chemotherapy. P-glycoprotein (P-gp) encoded by the *ABCB1/MDR1* gene is an important mechanism of MDR and is upregulated in many clinically resistant and refractory tumors (Kuete et al., 2015a). Overexpression of P-gp is causatively linked to accelerated efflux of chemotherapeutic agents (Kadioglu et al., 2016b) such as doxorubicin, daunorubicin, vincristine, etoposide, colchicine, camptothecins, and methotrexate (Dean, 2009). For instance, P-gp-overexpressing leukemia cells involve doxorubicin resistance compared to the sensitive subline (Kadioglu et al., 2016a). BCRP is involved in the efflux of mitoxantrone, topotecan, doxorubicin, daunorubicin, irinotecan, imatinib, and methotrexate (Dean, 2009).

Oridonin is a diterpenoid isolated from *Rabdosia rubescens* and reveals anticancer activity *in vitro* and *in vivo* (Xiao et al., 2016; Lu et al., 2017; Yao et al., 2017), but its mode of action and effect on drug resistance have not been well studied. *R. rubescens* inhibited breast cancer growth and angiogenesis (Sartippour et al., 2005) and overcame drug resistance in ADR/MCF-7 breast cancer cells by increasing doxorubicin accumulation (Li et al., 2013). Therefore, it is reasonable to investigate oridonin’s mode of action on MDR in more detail.

In this study, we analyzed molecular factors determining the response of tumor cells to oridonin. Various drug resistance mechanisms were investigated. We addressed three main questions:

(1) Is oridonin able to bypass resistance caused by different mechanisms such as P-gp, EGFR, p53, and BCRP? Moreover, can oridonin selectively target tumor cells rather than normal cells? To address these questions, we performed cytotoxicity assays.
(2) Are there other determinants predicting sensitivity or resistance of cancer cells to oridonin? For this reason, we performed COMPARE- and hierarchical cluster analyses of transcriptome-wide mRNA expression profiles of cancer cells.
(3) Can oridonin interact with EGFR pathway proteins? To answer this question, we applied molecular docking, MD, and Western blot.

## Materials and Methods

### Cell Lines

CCRF-CEM leukemia cells were cultured as previously described (Efferth et al., 2003b). Drug resistance of P-gp/*MDR1/ABCB1*-overexpressing CEM/ADR5000 cells was maintained in 5000 ng/mL doxorubicin (Kimmig et al., 1990). Breast cancer cells transduced with a control vector (MDA-MB-231-pcDNA3) or with cDNA for the breast cancer resistance protein *BCRP/ABCG2* (MDA-MB-231-BCRP clone 23) were generated and maintained as reported (Doyle et al., 1998). The mRNA expression of *MDR1* and *BCRP* in the resistant cell lines has been reported (Efferth et al., 2003a; Gillet et al., 2004). Human wild-type HCT116 colon cancer cells (p53^+/+^) as well as knockout clones (p53^-/-^) derived by homologous recombination (Bunz et al., 1998) were a generous gift from Dr. B. Vogelstein and H. Hermeking (Howard Hughes Medical Institute, Baltimore, MD, United States) and cultured as described (Bunz et al., 1998).

Human GBM U87MG cells transduced with an expression vector harboring an *EGFR* gene with a deletion of exons 2–7 (U87MG.ΔEGFR) has been previously reported (Huang et al., 1997). Transduced and non-transduced cell lines were kindly provided by Dr. W. K. Cavenee (Ludwig Institute for Cancer Research, San Diego, CA, United States). Human HepG2 hepatocellular carcinoma cells and AML12 normal hepatocytes were obtained from the American Type Cell Culture Collection (ATCC, United States).

### Resazurin Cell Growth Inhibition Assay

The resazurin (Promega, Mannheim, Germany) reduction assay (O’Brien et al., 2000) was used to assess the cytotoxicity as previously described (Kuete et al., 2015b, 2016). Each assay was conducted at least three times, with two replicates each. Cell viability was evaluated based on a comparison with untreated cells. IC_50_ values were determined as concentrations required to inhibit 50% of cell proliferation and were calculated from a calibration curve by linear regression using Microsoft Excel.

### Molecular Docking

The protocol for molecular docking was previously reported by us (Kadioglu et al., 2016b). An X-ray crystallography-based structure of wild-type Akt2 kinase domain (PDB ID: 3E87), EGFR (PDB ID: 1M17), mTOR (PDB ID: 4JSP), STAT3 DNA-binding domain, and VEGFR1 (PDB ID: 3HNG) were obtained from Protein Data Bank^1^. Homology model of STAT3 DNA-binding domain was created by us using MODELLER 9.11 (Fiser and Sali, 2003; Venkatachalam et al., 2003) and a Swiss-MODEL structure assessment tool^2^ based on the wild-type structure (PDB ID: 1BG1) as template. In order to assess the effect of an Akt2 mutation and EGFR mutation on oridonin binding, one point mutation-R274H on Akt2 was selected which has been shown to be critical for phosphatase resistance and keeping the phosphorylated status on Akt2 (Chan et al., 2011) and one point mutation-T790M on EGFR which has been shown to cause resistance to EGFR tyrosine kinase inhibitors (Zhou et al., 2018). Homology model of R274H mutant Akt2 kinase domain was created in the same manner by using wild-type Akt2 kinase domain as template. T790M-mutant EGFR structure is available in PDB database (PDB ID: 5XDK). A grid box was then constructed to define docking spaces in each protein according to their pharmacophores. Docking parameters were set to 250 runs and 2,500,000 energy evaluations for each cycle. Docking was performed three times independently by Autodock4 and with AutodockTools-1.5.7rc1 (Morris et al., 2009) using the Lamarckian Algorithm. The corresponding lowest binding energies and pKi were obtained from the docking log files (dlg). Mean ± SD of binding energies were calculated from three independent docking. Visual Molecular Dynamics (VMD) was used to depict the docking poses of oridonin and the inhibitors for each target protein.

### Molecular Dynamics

Lowest binding energy conformation of oridonin on Akt2 kinase domain prior to molecular docking analyses was picked to create ligand–protein complex structure for the MD simulations. QwikMD tool (Ribeiro et al., 2016) was used to perform 15 ns MD simulations after the equilibration of the protein–ligand complex. Stability of the docking pose was evaluated by root mean square deviation (RMSD) distance of the conformation throughout the MD simulation with the starting conformation. Total energy of the ligand–protein complex was calculated as well.

### Western Blot

In order to evaluate the effect of oridonin on EGFR pathway proteins and validate the *in silico* results, varying concentrations of oridonin (IC_50_/4, IC_50_/2, IC_50_, 2xIC_50_, and 4xIC_50_), determined after the cytotoxicity test on U87MG*.ΔEGFR* cell line, were applied in a similar way as described previously (Saeed et al., 2015). Briefly, 1 million cells per well were seeded in 12-well plate, next day treatment with oridonin was performed, total protein were extracted after 24 h. The following primary antibodies (Cell Signaling Technology, Frankfurt, Germany) were used: anti-rabbit EGFR, anti-rabbit phosphorylated EGFR (Tyr1068) (1:1000), anti-rabbit STAT3, anti-mouse phosphorylated STAT3 (Tyr705) (1:1000), anti-rabbit Akt, anti-rabbit phosphorylated Akt (Ser473) (1:1000), and anti-rabbit β-actin (1:2000).

### COMPARE and Hierarchical Cluster Analyses

The microarray-based mRNA expression values of genes of interest and log_10_IC_50_ values for oridonin of 49 tumor cell lines were selected from the NCI database^3^. The COMPARE analyses were performed to produce rank-ordered lists of genes expressed in the NCI cell lines. The methodology has been previously described in detail (Wosikowski et al., 1997). Briefly, every gene of the NCI microarray database was ranked for similarity of its mRNA expression to the log_10_IC_50_ values for oridonin based on Pearson’s rank correlation test. To derive COMPARE rankings, a scale index of correlations coefficients (*R*-values) was created. CIM miner software was used to perform the hierarchical clustering and heat map analysis^4^.

### Statistical Analyses

Results were represented as mean ± SD. Student’s *t*-test was performed in order to evaluate the statistical significance with two tails and unequal variance. Experiments with *p*-values lower than 0.05 were accepted as statistically significant.

## Results

### Response of Drug-Resistant Tumor Cell Lines Toward Oridonin

Cytotoxicity of oridonin and doxorubicin toward sensitive and drug-resistant cancer cell lines and normal cells were determined by the resazurin reduction assay (**Table 1**). The recorded IC_50_ values ranged from 1.65 (toward CCRF-CEM cells) to 34.68 μM (against HCT116P53^-/-^ cells) for oridonin and from 0.24 (toward CCRF-CEM cells) to 195.12 μM (against HCT116P53^-/-^ cells) for doxorubicin. The degree of resistance of resistant cells was calculated by dividing the IC_50_ value of this cell line by the IC_50_ value of the parental sensitive cells (**Table 1**). Oridonin was tested against multidrug-resistant P-gp (*MDR1*/*ABCB1*)-overexpressing CEM/ADR5000 cells and drug-sensitive parental CCRF-CEM cells using a resazurin assay. Although a weak cross-resistance of the CEM/ADR5000 cells was obtained (5.17-fold), this was much lower than that obtained with doxorubicin (975.60-fold). In another cell model for MDR, we compared the cytotoxicity of oridonin toward MDA-MB-231 cells transfected with *BCRP/ABCG2* and cells transfected with pcDNA control vector. The *BCRP* transfectants were 1.61-fold more resistant to oridonin than their sensitive counterparts. The activities of oridonin in knockout HCT116 (p53^-/-^) cells and their sensitive wild-type HCT116 (p53^+/+^) cells were also compared. *TP53*-knockout cells were cross-resistant to this compound than the *TP53* wild-type cells (degree of resistance: 1.92). However, the degree of resistance was slightly less resistant than that obtained with doxorubicin (2.84-fold). Interestingly, U87MG cells transfected with a deletion-activated *EGFR* cDNA were considerably more sensitive to oridonin than their wild-type counterpart (degree of resistance: 0.88). Normal AML10 hepatocytes were more resistant to oridonin than HepG2 hepatocellular carcinoma cells, indicating that the cytotoxic effects of oridonin may display tumor specificity at least to some extent.

**Table 1:** Cytotoxicity of oridonin and doxorubicin toward sensitive and drug-resistant cancer cell lines and normal cells as determined by the resazurin reduction assay.

Cell lines	Compounds, IC_50_ values in μM, and degree of resistance^a^ (in bracket)		
	Oridonin	Doxorubicin	Resistance mechanism
CCRF-CEM	1.65 ± 0.14	0.24 ± 0.02	
CEM/ADR5000	8.53 ± 0.77 (5.17)	195.12 ± 14.30 (975.60)	P-gp
MDA-MB231	6.06 ± 0.71	1.10 ± 0.01	
MDA-MB231/*BCRP*	9.74 ± 1.04 (1.61)	7.83 ± 0.01 (7.11)	BCRP
HCT116 (*p53^+/+^*)	18.03 ± 1.61	1.43 ± 0.02	
HCT116 (*p53^-/-^*)	34.68 ± 2.98 (1.92)	4.06 ± 0.04 (2.84)	p53
U87MG	17.37 ± 1.16	1.06 ± 0.03	
U87MG*.ΔEGFR*	15.34 ± 1.67 (0.88)	6.11 ± 0.04 (5.76)	EGFR
AML12	>109.76	>73.59	
HepG2	25.71 ± 2.11 (<0.23)	1.41 ± 0.12 (<0.04)	Tumor versus normal cells

*Mean values ± SD of each three independent experiments with each six parallel measurements are shown. ^a^The degree of resistance was determined as the ratio of IC_50_ value of the resistant/IC_50_-sensitive cell line. N.a., not applicable.*

### Molecular Docking and Molecular Dynamics

**FIGURE 1:**
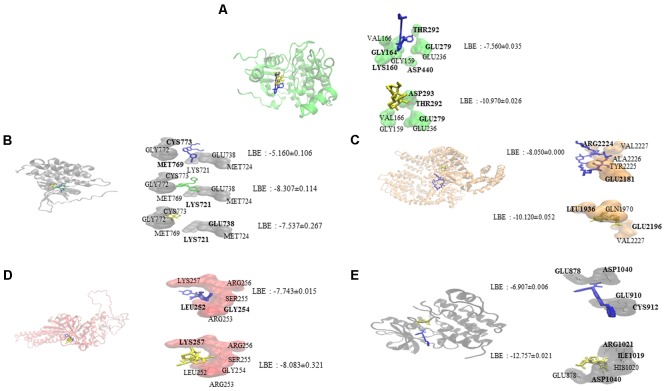
Molecular docking studies of oridonin and known inhibitors on proteins involved in EGFR signaling pathway. Proteins have been represented in new cartoon format with different colors, while oridonin was represented in yellow. **(A)** Docking poses in to the pharmacophore of Akt2 kinase domain (PDB code: 3E87 in green cartoon representation). GSK690693 was represented in blue. **(B)** Docking poses in to the pharmacophore of EGFR tyrosine kinase domain (PDB code: 1M17 in gray cartoon representation). Gefitinib was represented in green and erlotinib was represented in blue. **(C)** Docking poses in to the pharmacophore of mTOR (PDB code: 4JSP in orange representation). Sirolimus was represented in blue. **(D)** Docking poses in to the pharmacophore of STAT3 DNA-binding domain (homology model created by using the template PDB code: 1BG1 in pink cartoon representation). NSC74859 was represented in blue. **(E)** Docking poses in to the pharmacophore of VEGFR1 (PDB code: 3HNG in black cartoon representation). Axitinib was represented in blue.

Oridonin interacts with EGFR signaling pathway proteins, as can be seen in **Figure 1**. Comparable binding energies and docking poses were observed for Akt2 and STAT3 proteins with those of known inhibitors [GSK690693 (Heerding et al., 2008) for Akt2 and NSC74859 (Zhang et al., 2014) for STAT3]. ATP-binding domain of Akt2 consists of the following residues: Leu158, Val166, Ala179, Val213, Met229, Tyr231, Met282, Thr292, Phe294, Ala322, and Phe439 (Huang et al., 2003). Oridonin interacts with Val166 and forms hydrogen bond with Thr292 implying its inhibitory effect.

Oridonin can still bind with comparable binding energies on mutant Akt2 and EGFR as can be seen in **Table 2**. Interestingly, oridonin interacts with EGFR-T790M significantly stronger than to wild-type EGFR (-6.633 vs. -5.160 kcal/mol). pKi is significantly lower as well (14.283 vs. 166.310 μM). Gefitinib binds to EGFR-T790M significantly weaker than to wild-type EGFR (-7.773 vs. -8.307 kcal/mol).

**Table 2:** Comparison of binding energies of oridonin and known inhibitors on wild-type and mutant Akt2 and EGFR (LBE, kcal/mol; pKi, μM).

	EGFR wt		EGFR-T790M		LBE *p*-value	pKi *p*-value
	LBE	pKi	LBE	pKi		
Oridonin	-5.160 ± 0.106	166.310 ± 30.323	-6.633 ± 0.202	14.283 ± 5.300	<0.05	<0.05
Erlotinib	-7.537 ± 0.267	3.183 ± 1.222	-7.547 ± 0.371	3.293 ± 1.645	>0.05	>0.05
Gefitinib	-8.307 ± 0.114	0.820 ± 0.161	-7.773 ± 0.196	2.070 ± 0.615	<0.05	>0.05

	**Akt2 wt**		**Akt2-R274H**		**LBE *p*-value**	**pKi *p*-value**
	**LBE**	**pKi**	**LBE**	**pKi**		

Oridonin	-7.560 ± 0.035	2.873 ± 0.188	-7.253 ± 0.023	4.830 ± 0.166	<0.05	<0.05
Gsk690693	-10.970 ± 0.026	0.091 ± 0.004	-10.930 ± 0.010	0.097 ± 0.002	>0.05	>0.05

**FIGURE 2:**
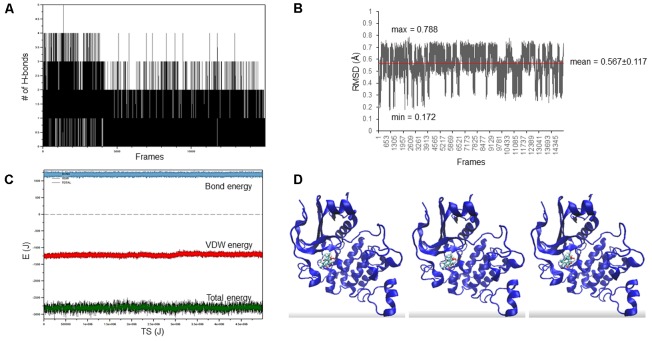
Oridonin–Akt2 kinase domain MD simulation. **(A)** Number of H-bonds between oridonin and Akt2. **(B)** RMSD of oridonin aligned with the LBE conformation acquired after molecular docking calculations. **(C)** Van der Waals, total energy, bond energy of oridonin–Akt2 complex. **(D)** Representative screenshots from the MD simulation.

The docking pose of oridonin on Akt2 kinase domain was used as starting conformation for the MD simulation. As can be seen in **Figure 2**, the LBE conformation of oridonin was stable, since the RMSD value was below 1 Å (0.567 ± 0.117) throughout 15 ns simulation.

### Western Blot

**FIGURE 3:**
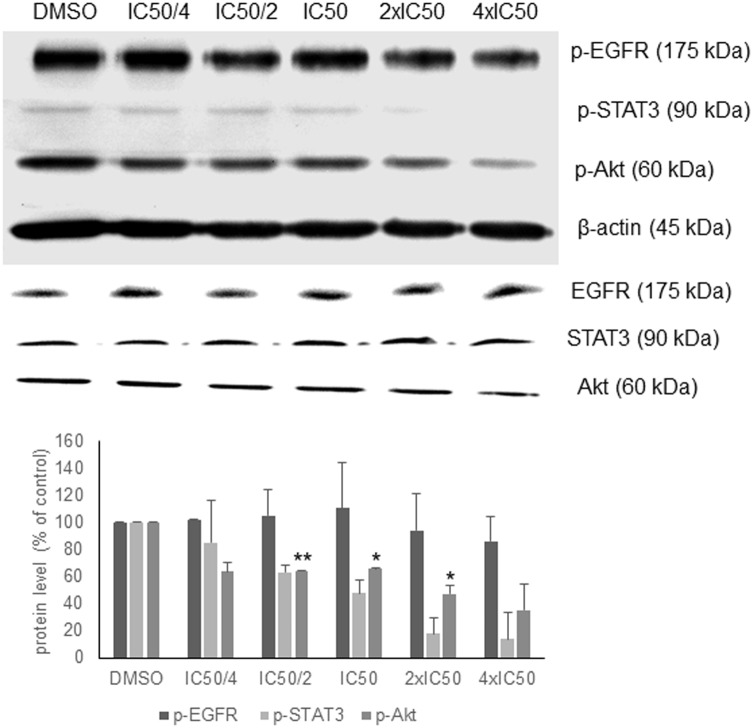
Western blot analysis of oridonin on EGFR pathway proteins. The effects of oridonin on phosphorylation of ΔEGFR, STAT3, and Akt were evaluated. Bands were normalized to β-actin in order to obtain numerical values (mean ± SEM of three independent experiments). Total EGFR, STAT3, and Akt protein levels are also shown. A representative blot is shown and statistical analysis was done by paired Student’s *t*-test. ^∗∗^*p* < 0.01, *^∗^p* < 0.05.

In order to validate the *in silico* analyses, the phosphorylation status of EGFR signaling proteins as parameter of their activation during signal transduction was investigated. Oridonin revealed a dose-dependent inhibition of Akt and STAT3 phosphorylation supporting the *in silico* analyses, but no change in EGFR phosphorylation was observed (**Figure 3**). There was no change at the total Akt, EGFR, and STAT3 protein levels.

### Pharmacogenomics

We investigated the transcriptome-wide RNA expression using COMPARE analysis and mined the database of the NCI by correlating the mRNA expression data with the log_10_IC_50_ values for oridonin. This is a hypothesis-generating bioinformatical approach allowing to find novel putative molecular determinants of cellular response to oridonin. The scale rankings of genes obtained by COMPARE computation were subjected to Pearson’s rank correlation tests. The thresholds for correlation coefficients were *R* > 0.50 for direct correlations and *R* < -0.50 for inverse correlations. As shown in **Table 3**, the identified genes can be assigned to different functional groups such as apoptosis regulation (*CARD8*, *ANXA5*), transcriptional and protein synthesis (*FUBP1*, *RFXAP*, *CELF2*, *TWF1*, *COQ9*), DNA repair and maintenance (*RAD51C*, *BLM*), signal transduction (*PRKXP1*, *SRPK1*, *GNG12*, *IL6ST*, *PTPRK*, *MET*), cell cycle regulation (*MND1*, *PDS5B*, *PHF8*), transport functions (*ABCC5*), cellular energy regulation (*PKM2*), cell adhesion (*LAMB1*, *ADAM9*), and ubiquitination (*USP44*).

**Table 3:** Correlation of constitutive mRNA expression of genes identified by COMPARE analyses with log_10_IC_50_ values of oridonin.

COMPARE coefficient	Experiment ID	GB accession	Gene symbol	Name	Function
0.717	GC158023	AI652861	*CELF2*	CUGBP, Elav-like family member 2	Pre-mRNA alternative splicing, mRNA translation, and stability
0.705	GC80864	AI983986	*PRKXP1*	Protein kinase, X-linked, pseudogene 1	Protein kinase homologous to *Drosophila* DC2 kinase pseudogene 1
0.681	GC32017	AB020630	*PPP1R16B*	Protein phosphatase 1, regulatory (inhibitor) subunit 16B	Regulator of pulmonary endothelial cell (EC) barrier function
0.665	GC186446	NM_014959	*CARD8*	Caspase recruitment domain family, member 8	Inhibitor of NF-κ-B activation
0.664	GC31918	AF029670	*RAD51C*	RAD51 homolog C (*S. cerevisiae*)	Homologous recombination (HR) DNA repair pathway
0.662	GC35837	Z25535	*NUP153*	Nucleoporin 153 kDa	DNA-binding subunit of the nuclear pore complex (NPC)
0.662	GC78746	AI927080	*HDHD2*	Haloacid dehalogenase-like hydrolase domain containing 2	Hydrolase activity
0.656	GC163915	AL036840	*FUBP1*	Far upstream element (FUSE)-binding protein 1	Regulator of MYC expression by binding to a single-stranded far-upstream element (FUSE) upstream of the MYC promoter
0.655	GC30354	AB023139	*KIAA0922*	KIAA0922	Not available
0.654	GC169231	AW249934	*PHF8*	PHD finger protein 8	Cell cycle progression, rDNA transcription, and brain development
0.653	GC100782	X78817	*ARHGAP4*	Rho GTPase activating protein 4	Inhibitor of stress fiber organization
0.652	GC172537	BC006312	*CROCCL1*	Ciliary rootlet coiled-coil, rootletin-like 1	Not available
0.652	GC182282	NM_003137	*SRPK1*	SRSF protein kinase 1	Phosphorylation of SR splicing factors and regulation of splicing
0.651	GC75737	AI815763	*ABCC5*	ATP-binding cassette, sub-family C (CFTR/MRP), member 5	Multispecific organic anion pump for nucleotide analogs
0.65	GC46177	AA542845	*MND1*	Meiotic nuclear divisions 1 homolog (*S. cerevisiae*)	Homologous chromosome pairing, cross-over, and intragenic recombination during meiosis
0.648	GC152371	AF061734	*DTNBP1*	Dystrobrevin-binding protein 1	Biogenesis of lysosome-related organelles (LRO), such as platelet dense granules and melanosomes
0.647	GC37727	U42031	*FKBP5*	FK506-binding protein 5	Complexation with heterooligomeric progesterone receptor, HSP90, and TEBP
0.645	GC44551	AA448146	*USP44*	Ubiquitin-specific peptidase 44	Deubiquitinase that prevents premature anaphase onset in the spindle assembly checkpoint
0.642	GC33552	U39817	*BLM*	Bloom syndrome, RecQ helicase-like	DNA replication and repair
0.64	GC72681	AI742868	*RFXAP*	Regulatory factor X-associated protein	Part of the RFX complex that binds to the X-box of MHC II promoters
-0.692	GC18026	AA004918	*LAMB1*	Laminin, β1	Attachment, migration, and organization of cells into tissues during embryonic development
-0.667	GC16842	AA025336	*SPATS2L*	Spermatogenesis-associated, serine-rich 2-like	Not available
-0.659	GC9768	AA047421	*GNG12*	Guanine nucleotide-binding protein (G protein), γ12	Modulator or transducer in transmembrane signaling systems
-0.645	GC18754	AA036724	*CAV2*	Caveolin 2	Regulation of G-protein α-subunits
-0.642	GC10289	AA053017	*ANXA5*	Annexin A5	Bloodvanticoagulant protein that inhibits the thromboplastin-specific complex
-0.64	GC9921	AA045041	*TWF1*	Twinfilin, actin-binding protein, homolog 1 (*Drosophila*)	Inhibitor of actin polymerization likely by sequestering G-actin
-0.623	GC18611	AA034024	*RAI14*	Retinoic acid induced 14	Not available
-0.617	GC15762	W47533	*ADAM9*	ADAM metallopeptidase domain 9	Cell–cell or cell–matrix interactions
-0.615	GC13860	H85457	*IL6ST*	Interleukin 6 signal transducer (gp130, oncostatin M receptor)	Signal-transducing molecule
-0.613	GC10564	T72607	*PDS5B*	PDS5, regulator of cohesion maintenance, homolog B (*S. cerevisiae*)	Regulator of sister chromatid cohesion in mitosis which stabilizes cohesin complex association with chromatin
-0.612	GC18739	AA035170	*TICAM2*	Toll-like receptor adaptor molecule 2	Regulator of the MYD88-independent pathway during the innate immune response to LPS
-0.607	GC9920	AA045034	*OSTM1*	Osteopetrosis-associated transmembrane protein 1	Osteoclast and melanocyte maturation and function
-0.605	GC174276	BE908217	*ANXA2*	Annexin A2	Calcium-regulated membrane-binding protein
-0.599	GC180243	NM_000445	*PLEC*	Plectin	Linker of intermediate filaments with microtubules and microfilaments and anchor of intermediate filaments to desmosomes or hemidesmosomes
-0.599	GC33491	L77886	*PTPRK*	Protein tyrosine phosphatase, receptor type, K	Negative regulator of EGFR signaling pathway
-0.597	GC89723	M26252	*PKM2*	Pyruvate kinase, muscle	Transfer of a phosphoryl group from phosphoenolpyruvate (PEP) to ADP to generate ATP
-0.59	GC13104	R97218	*MET*	Met proto-oncogene (hepatocyte growth factor receptor)	Signal transducer from the extracellular matrix into the cytoplasm
-0.585	GC187142	NM_016639	*TNFRSF12A*	Tumor necrosis factor receptor superfamily, member 12A	Angiogenesis and proliferation of endothelial cells
-0.584	GC14757	N62737	*MFAP3L*	Microfibrillar-associated protein 3-like	Not available
-0.581	GC18658	AA034910	*COQ9*	Coenzyme Q9 homolog (*S. cerevisiae*)	Biosynthesis of coenzyme Q

**FIGURE 4:**
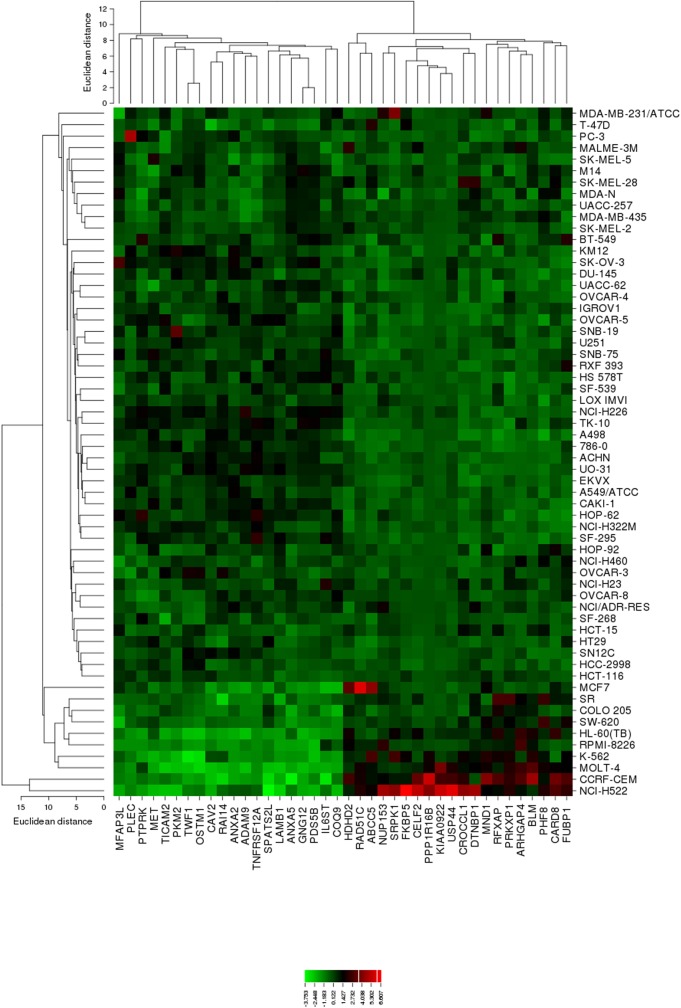
Heat map obtained by hierarchical cluster analysis from microarray-based mRNA expression profiles of the genes shown in **Table 3** correlating with cellular responsiveness to oridonin. The analysis shows the clustering of 49 NCI tumor cell lines.

The mRNA expression values of all NCI cell lines for the genes listed in **Table 3** were subsequently subjected to agglomerative hierarchical cluster analysis, in order to find out, whether clusters of cell lines could be identified with similar behavior after exposure to oridonin. The dendrogram of the cluster analysis showed three clusters (**Figure 4**). As a next step, the log_10_IC_50_ values for oridonin, which were not included in the cluster analysis, were assigned to the corresponding position of the cell lines in the cluster tree. The distribution among the clusters was significantly different from each other as determined by Chi-square test (*p*-value = 0.0014). Cluster 1 contained in its majority of cell lines resistant to oridonin, whereas Cluster 3 contained in its majority sensitive ones (Cluster 1: 17 resistant and 7 sensitive; Cluster 2: 8 resistant and 8 sensitive; Cluster 3: 0 resistant and 9 sensitive).

## Discussion

Oridonin is a diterpenoid isolated from *R. rubescens* with previously reported anticancer activity. It targets PI3K/Akt pathway causing G2/M arrest in prostate cancer cells (Lu et al., 2017). Oridonin also targets Notch signaling leading to inhibition of breast cancer progression (Xia et al., 2017). According to the literature, 4 μg/mL or 10 μM is the upper IC_50_ limit considered for a promising cytotoxic compound after incubation for 48 and 72 h (Boik, 2001; Brahemi et al., 2010; Kuete and Efferth, 2015). In the present work, oridonin displayed IC_50_ values (<10 μM) within this threshold value toward four tested cancer cell lines as determined by resazurin assay. These data show the antiproliferative potential of oridonin against drug-sensitive and -resistant cancer cell lines since identification of compounds able to overcome MDR is an attractive strategy in drug research (Efferth, 2001; Gottesman and Ling, 2006; Gillet et al., 2007). Interestingly, the resistant U87MG.ΔEGFR cells were even more sensitive to oridonin than their corresponding sensitive counterparts (U87MG cells). If cross-resistance was obtained, the degree or resistance was lower in all cases than that of the reference compound, doxorubicin. This suggests that oridonin could be explored further to develop a cytototoxic drug to combat MDR phenotypes.

Overexpression of P-gp, a broad spectrum drug transporter, leads to the efficient extrusion of a large number of established anticancer drugs and cytotoxic natural products out of cancer cells. This is the main reason, why tumors with P-gp overexpression exert a MDR phenotype limiting the success of established drugs. Therefore, it was a pleasing result that the expression of P-gp/*MDR1* in the NCI cell line panel did not correlate with cellular response to oridonin, which implies that P-gp does not confer resistance to oridonin. In addition, multidrug-resistant CEM/ADR5000 cells with overexpression of various ABC transporters including P-gp/*MDR1* (400-fold) (Kadioglu et al., 2016a) revealing high degrees of resistance to well-known anticancer drugs such as doxorubicin (1036-fold), vincristine (613-fold), docetaxel (435-fold), and many others (Efferth et al., 2008) were even slightly more sensitive to oridonin than the parental, wild-type, drug-sensitive CCRF-CEM tumor cells. It can be speculated that oridonin successfully kills otherwise unresponsive, multidrug-resistant tumors.

Oridonin revealed comparable binding energies to EGFR pathway proteins as the known inhibitors. It shares the same docking pose with GSK690693 on Akt2 and NSC74859 on STAT3, implying the inhibitory potential of oridonin toward Akt2 and STAT3. MD study revealed that oridonin docking pose on Akt2 is stable throughout the simulation with a relatively small RMSD deviation (<1 Angström). In order to validate the *in silico* findings and further evaluate the mode of action of oridonin, western blot experiments for the EGFR pathway proteins regarding their phosphorylation status upon oridonin treatment were performed. Results implied that cytotoxicity of oridonin is dependent on the EGFR pathway influence.

Various other resistance factors in addition to EGFR and P-gp determine the success rate of chemotherapy. In order to achieve a deeper understanding of drug response determinant mechanisms, microarray technology is widely used. This methodology is especially helpful to identify potential mechanisms of novel, still incompletely understood cytotoxic compounds. For this purpose, we performed COMPARE and hierarchical cluster analyses of transcriptome-wide, microarray-based mRNA expression of the NCI cell line panel. The expression of the genes identified via COMPARE analyses determined cellular response to oridonin in this panel of cell lines.

Despite P-gp expression was not correlated to oridonin resistance, our COMPARE analysis revealed that another member of ABC superfamily, i.e., ABCC5, was a molecular determinant to mediate resistance to oridonin in the NCI cancer cell line panel. In this context, it is worth to mention that a member of the ABC sub-family C, ABCC1 (multidrug protein 1, MRP1), which is known to confer MDR phenotype differs from that one caused by P-gp (ABCB1/MDR1) and BCRP/ABCG2 (Efferth, 2001).

In conclusion, oridonin targeted various resistance mechanisms and inhibited Akt2 and STAT3 phosphorylation. The resistance and sensitivity genes identified may be helpful for the development of personalized therapy approaches, as their expression in an individual patient may suggest potentially successful oridonin treatment in the future. However, further preclinical and clinical studies are required to assess the therapeutic potential of oridonin for cancer therapy.

## Author Contributions

TE conceived the study. OK performed the *in silico* experiments. MS contributed to the *in silico* experiments. OK, MS, HG, and TE wrote the manuscript. OK, MS, and VK performed the *in vitro* experiments. All the authors read the manuscript.

## Conflict of Interest Statement

The authors declare that the research was conducted in the absence of any commercial or financial relationships that could be construed as a potential conflict of interest.

## Footnotes

1 http://www.rcsb.org/pdb

2 http://swissmodel.expasy.org/

3 http://dtp.nci.nih.gov

4 https://discover.nci.nih.gov/cimminer/

## ABBREVIATIONS

GBM: glioblastoma multiforme
LBE: lowest binding energy
MDR: multidrug resistance
MD: molecular dynamics
pKi: predicted inhibition constant.

## References

[B1] Boik J. (2001). *Natural Compounds in Cancer Therapy.* Princeton, MN: Oregon Medical Press.

[B2] Brahemi G. Kona F. R. Fiasella A. Buac D. Soukupova J. Brancale A. (2010). Exploring the structural requirements for inhibition of the ubiquitin E3 ligase breast cancer associated protein 2 (BCA2) as a treatment for breast cancer. *J. Med. Chem.* 53 2757–2765. 10.1021/jm901757t 20222671 PMC2848690

[B3] Bunz F. Dutriaux A. Lengauer C. Waldman T. Zhou S. Brown J. P. (1998). Requirement for p53 and p21 to sustain G2 arrest after DNA damage. *Science* 282 1497–1501. 10.1126/science.282.5393.1497 9822382

[B4] Chan T. O. Zhang J. Rodeck U. Pascal J. M. Armen R. S. Spring M. (2011). Resistance of Akt kinases to dephosphorylation through ATP-dependent conformational plasticity. *Proc. Natl. Acad. Sci. U.S.A.* 108 E1120–E1127. 10.1073/pnas.1109879108 22031698 PMC3219155

[B5] Dean M. (2009). ABC transporters, drug resistance, and cancer stem cells. *J. Mammary Gland Biol. Neoplasia* 14 3–9. 10.1007/s10911-009-9109-9 19224345

[B6] Doyle L. A. Yang W. Abruzzo L. V. Krogmann T. Gao Y. Rishi A. K. (1998). A multidrug resistance transporter from human MCF-7 breast cancer cells. *Proc. Natl. Acad. Sci. U.S.A.* 95 15665–15670. 10.1073/pnas.95.26.15665 9861027 PMC28101

[B7] Efferth T. (2001). The human ATP-binding cassette transporter genes: from the bench to the bedside. *Curr. Mol. Med.* 1 45–65. 10.2174/1566524013364194 11899242

[B8] Efferth T. (2012). Signal transduction pathways of the epidermal growth factor receptor in colorectal cancer and their inhibition by small molecules. *Curr. Med. Chem.* 19 5735–5744. 10.2174/092986712803988884 23033949

[B9] Efferth T. (2017). Cancer combination therapy of the sesquiterpenoid artesunate and the selective EGFR-tyrosine kinase inhibitor erlotinib. *Phytomedicine* 37 58–61. 10.1016/j.phymed.2017.11.003 29174651

[B10] Efferth T. Gebhart E. Ross D. D. Sauerbrey A. (2003a). Identification of gene expression profiles predicting tumor cell response to L-alanosine. *Biochem. Pharmacol.* 66 613–621. 12906926 10.1016/s0006-2952(03)00341-1

[B11] Efferth T. Konkimalla V. B. Wang Y. F. Sauerbrey A. Meinhardt S. Zintl F. (2008). Prediction of broad spectrum resistance of tumors towards anticancer drugs. *Clin. Cancer Res.* 14 2405–2412. 10.1158/1078-0432.CCR-07-4525 18413831

[B12] Efferth T. Sauerbrey A. Olbrich A. Gebhart E. Rauch P. Weber H. O. (2003b). Molecular modes of action of artesunate in tumor cell lines. *Mol. Pharmacol.* 64 382–394. 12869643 10.1124/mol.64.2.382

[B13] Fiser A. Sali A. (2003). Modeller: generation and refinement of homology-based protein structure models. *Methods Enzymol.* 374 461–491. 10.1016/S0076-6879(03)74020-8 14696385

[B14] Furnari F. B. Fenton T. Bachoo R. M. Mukasa A. Stommel J. M. Stegh A. (2007). Malignant astrocytic glioma: genetics, biology, and paths to treatment. *Genes Dev.* 21 2683–2710. 10.1101/gad.1596707 17974913

[B15] Gallorini M. Cataldi A. Di Giacomo V. (2012). Cyclin-dependent kinase modulators and cancer therapy. *BioDrugs* 26 377–391. 10.2165/11634060-000000000-00000 22928661

[B16] Garcia-Carbonero R. Carnero A. Paz-Ares L. (2013). Inhibition of HSP90 molecular chaperones: moving into the clinic. *Lancet Oncol.* 14 e358–e369. 10.1016/S1470-2045(13)70169-4 23896275

[B17] Gillet J. Efferth T. Steinbach D. Hamels J. De Longueville F. Bertholet V. (2004). Microarray-based detection of multidrug resistance in human tumor cells by expression profiling of ATP-binding cassette transporter genes. *Cancer Res.* 64 8987–8993. 10.1158/0008-5472.CAN-04-1978 15604263

[B18] Gillet J. P. Efferth T. Remacle J. (2007). Chemotherapy-induced resistance by ATP-binding cassette transporter genes. *Biochim. Biophys. Acta* 1775 237–262. 10.1016/j.bbcan.2007.05.002 17572300

[B19] Gottesman M. M. Ling V. (2006). The molecular basis of multidrug resistance in cancer: the early years of P-glycoprotein research. *FEBS Lett.* 580 998–1009. 10.1016/j.febslet.2005.12.060 16405967

[B20] Guo Y. Ding Y. Y. Zhang T. An H. L. (2016). Sinapine reverses multi-drug resistance in MCF-7/dox cancer cells by downregulating FGFR4/FRS2 alpha-ERK1/2 pathway-mediated NF-kappa B activation. *Phytomedicine* 23 267–273. 10.1016/j.phymed.2015.12.017 26969380

[B21] Heerding D. A. Rhodes N. Leber J. D. Clark T. J. Keenan R. M. Lafrance L. V. (2008). Identification of 4-(2-(4-amino-125-oxadiazol-3-yl)-1-ethyl-7-{[(3S)-3-piperidinylmethyl]oxy}-1H- imidazo[45-c]pyridin-4-yl)-2-methyl-3-butyn-2-ol (GSK690693), a novel inhibitor of AKT kinase. *J. Med. Chem.* 51 5663–5679. 10.1021/jm8004527 18800763

[B22] Heimberger A. B. Hlatky R. Suki D. Yang D. Weinberg J. Gilbelbrt M. (2005). Prognostic effect of epidermal growth factor receptor and EGFRvIII in glioblastoma multiforme patients. *Clin. Cancer Res.* 11 1462–1466. 10.1158/1078-0432.CCR-04-1737 15746047

[B23] Huang H. S. Nagane M. Klingbeil C. K. Lin H. Nishikawa R. Ji X. D. (1997). The enhanced tumorigenic activity of a mutant epidermal growth factor receptor common in human cancers is mediated by threshold levels of constitutive tyrosine phosphorylation and unattenuated signaling. *J. Biol. Chem.* 272 2927–2935. 10.1074/jbc.272.5.2927 9006938

[B24] Huang X. Begley M. Morgenstern K. A. Gu Y. Rose P. Zhao H. (2003). Crystal structure of an inactive Akt2 kinase domain. *Structure* 11 21–30. 10.1016/S0969-2126(02)00937-1 12517337

[B25] Kadioglu O. Cao J. Kosyakova N. Mrasek K. Liehr T. Efferth T. (2016a). Genomic and transcriptomic profiling of resistant CEM/ADR-5000 and sensitive CCRF-CEM leukaemia cells for unravelling the full complexity of multi-factorial multidrug resistance. *Sci. Rep.* 6:36754. 10.1038/srep36754 27824156 PMC5099876

[B26] Kadioglu O. Cao J. Saeed M. E. Greten H. J. Efferth T. (2015). Targeting epidermal growth factor receptors and downstream signaling pathways in cancer by phytochemicals. *Target Oncol.* 10 337–353. 10.1007/s11523-014-0339-4 25410594

[B27] Kadioglu O. Saeed M. E. M. Valoti M. Frosini M. Sgaragli G. Efferth T. (2016b). Interactions of human P-glycoprotein transport substrates and inhibitors at the drug binding domain: Functional and molecular docking analyses. *Biochem. Pharmacol.* 104 42–51. 10.1016/j.bcp.2016.01.014 26807479

[B28] Khoury K. Domling A. (2012). P53 mdm2 inhibitors. *Curr. Pharm. Des.* 18 4668–4678. 10.2174/138161212802651580 22650254 PMC3719986

[B29] Kimmig A. Gekeler V. Neumann M. Frese G. Handgretinger R. Kardos G. (1990). Susceptibility of multidrug-resistant human leukemia cell lines to human interleukin 2-activated killer cells. *Cancer Res.* 50 6793–6799. 1698543

[B30] Kuete V. Efferth T. (2015). African flora has the potential to fight multidrug resistance of cancer. *Biomed Res. Int.* 2015:914813. 10.1155/2015/914813 25961047 PMC4413252

[B31] Kuete V. Fouotsa H. Mbaveng A. T. Wiench B. Nkengfack A. E. Efferth T. (2015a). Cytotoxicity of a naturally occurring furoquinoline alkaloid and four acridone alkaloids towards multi-factorial drug-resistant cancer cells. *Phytomedicine* 22 946–951. 10.1016/j.phymed.2015.07.002 26321744

[B32] Kuete V. Mbaveng A. T. Nono E. C. Simo C. C. Zeino M. Nkengfack A. E. (2016). Cytotoxicity of seven naturally occurring phenolic compounds towards multi-factorial drug-resistant cancer cells. *Phytomedicine* 23 856–863. 10.1016/j.phymed.2016.04.007 27288921

[B33] Kuete V. Sandjo L. P. Mbaveng A. T. Zeino M. Efferth T. (2015b). Cytotoxicity of compounds from *Xylopia aethiopica* towards multi-factorial drug-resistant cancer cells. *Phytomedicine* 22 1247–1254. 10.1016/j.phymed.2015.10.008 26655407

[B34] Lacaille-Dubois M. A. Wagner H. (2017). New perspectives for natural triterpene glycosides as potential adjuvants. *Phytomedicine* 37 49–57. 10.1016/j.phymed.2017.10.019 29239784

[B35] Leber M. F. Efferth T. (2009). Molecular principles of cancer invasion and metastasis (review). *Int. J. Oncol.* 34 881–895. 19287945 10.3892/ijo_00000214

[B36] Li F. Fan J. Wu Z. Liu R. Y. Guo L. Dong Z. (2013). Reversal effects of Rabdosia rubescens extract on multidrug resistance of MCF-7/Adr cells in vitro. *Pharm. Biol.* 51 1196–1203. 10.3109/13880209.2013.784342 23777360

[B37] Liu M. Z. Yang Y. Wang C. Sun L. D. Mei C. Z. Yao W. T. (2010). The effect of epidermal growth factor receptor variant III on glioma cell migration by stimulating ERK phosphorylation through the focal adhesion kinase signaling pathway. *Arch. Biochem. Biophys.* 502 89–95. 10.1016/j.abb.2010.07.014 20650261

[B38] Lu J. Chen X. Qu S. Yao B. Xu Y. Wu J. (2017). Oridonin induces G2/M cell cycle arrest and apoptosis via the PI3K/Akt signaling pathway in hormone-independent prostate cancer cells. *Oncol. Lett.* 13 2838–2846. 10.3892/ol.2017.5751 28454475 PMC5403405

[B39] Morris G. M. Huey R. Lindstrom W. Sanner M. F. Belew R. K. Goodsell D. S. (2009). AutoDock4 and AutoDockTools4: automated docking with selective receptor flexibility. *J. Comput. Chem.* 30 2785–2791. 10.1002/jcc.21256 19399780 PMC2760638

[B40] Muller P. A. Vousden K. H. (2013). p53 mutations in cancer. *Nat. Cell Biol.* 15 2–8. 10.1038/ncb2641 23263379

[B41] Nankar R. Prabhakar P. K. Doble M. (2017). Hybrid drug combination: combination of ferulic acid and metformin as anti-diabetic therapy. *Phytomedicine* 37 10–13. 10.1016/j.phymed.2017.10.015 29126698

[B42] Nankar R. P. Doble M. (2017). Hybrid drug combination: anti-diabetic treatment of type 2 diabetic Wistar rats with combination of ellagic acid and pioglitazone. *Phytomedicine* 37 4–9. 10.1016/j.phymed.2017.10.014 29103827

[B43] Natarajan K. Xie Y. Baer M. R. Ross D. D. (2012). Role of breast cancer resistance protein (BCRP/ABCG2) in cancer drug resistance. *Biochem. Pharmacol.* 83 1084–1103. 10.1016/j.bcp.2012.01.002 22248732 PMC3307098

[B44] Newman D. J. Cragg G. M. (2007). Natural products as sources of new drugs over the last 25 years. *J. Nat. Prod.* 70 461–477. 10.1021/np068054v 17309302

[B45] O’Brien J. Wilson I. Orton T. Pognan F. (2000). Investigation of the Alamar Blue (resazurin) fluorescent dye for the assessment of mammalian cell cytotoxicity. *Eur. J. Biochem.* 267 5421–5426. 10.1046/j.1432-1327.2000.01606.x 10951200

[B46] Reis M. A. Ahmed O. B. Spengler G. Molnar J. Lage H. Ferreira M. J. U. (2016). Jatrophane diterpenes and cancer multidrug resistance-ABCB1 efflux modulation and selective cell death induction. *Phytomedicine* 23 968–978. 10.1016/j.phymed.2016.05.007 27387405

[B47] Ribeiro J. V. Bernardi R. C. Rudack T. Stone J. E. Phillips J. C. Freddolino P. L. (2016). QwikMD - Integrative molecular dynamics toolkit for novices and experts. *Sci. Rep.* 6:26536. 10.1038/srep26536 27216779 PMC4877583

[B48] Saeed M. Jacob S. Sandjo L. P. Sugimoto Y. Khalid H. E. Opatz T. (2015). Cytotoxicity of the sesquiterpene lactones neoambrosin and damsin from *Ambrosia maritima* against multidrug-resistant cancer cells. *Front. Pharmacol.* 6:267. 10.3389/fphar.2015.00267 26617519 PMC4637410

[B49] Sartippour M. R. Seeram N. P. Heber D. Hardy M. Norris A. Lu Q. (2005). Rabdosia rubescens inhibits breast cancer growth and angiogenesis. *Int. J. Oncol.* 26 121–127. 10.3892/ijo.26.1.121 15586232

[B50] Schad F. Thronicke A. Merkle A. Matthes H. Steele M. L. (2017). Immune-related and adverse drug reactions to low versus high initial doses of *Viscum album* L. in cancer patients. *Phytomedicine* 36 54–58. 10.1016/j.phymed.2017.09.004 29157828

[B51] Spano D. Heck C. De Antonellis P. Christofori G. Zollo M. (2012). Molecular networks that regulate cancer metastasis. *Semin. Cancer Biol.* 22 234–249. 10.1016/j.semcancer.2012.03.006 22484561

[B52] Teng Y. N. Sheu M. J. Hsieh Y. W. Wang R. Y. Chiang Y. C. Hung C. C. (2016). beta-carotene reverses multidrug resistant cancer cells by selectively modulating human P-glycoprotein function. *Phytomedicine* 23 316–323. 10.1016/j.phymed.2016.01.008 26969385

[B53] Venkatachalam T. K. Qazi S. Samuel P. Uckun F. M. (2003). Inhibition of mast cell leukotriene release by thiourea derivatives. *Bioorg. Med. Chem. Lett.* 13 485–488. 10.1016/S0960-894X(02)00992-7 12565956

[B54] Wagner H. Efferth T. (2017). Introduction: Novel hybrid combinations containing synthetic or antibiotic drugs with plant-derived phenolic or terpenoid compounds. *Phytomedicine* 37 1–3. 10.1016/j.phymed.2017.10.020 29174652

[B55] Walkinshaw D. R. Yang X. J. (2008). Histone deacetylase inhibitors as novel anticancer therapeutics. *Curr. Oncol.* 15 237–243. 19008999 10.3747/co.v15i5.371 PMC2582508

[B56] Wang S. Zhao Y. Aguilar A. Bernard D. Yang C. Y. (2017). Targeting the MDM2-p53 protein-protein interaction for new cancer therapy: progress and challenges. *Cold Spring Harb. Perspect. Med.* 7:a026245. 10.1101/cshperspect.a026245 28270530 PMC5411684

[B57] Wosikowski K. Schuurhuis D. Johnson K. Paull K. D. Myers T. G. Weinstein J. N. (1997). Identification of epidermal growth factor receptor and c-erbB2 pathway inhibitors by correlation with gene expression patterns. *J. Natl. Cancer Inst.* 89 1505–1515. 10.1093/jnci/89.20.1505 9337347

[B58] Xia S. Zhang X. Li C. Guan H. (2017). Oridonin inhibits breast cancer growth and metastasis through blocking the Notch signaling. *Saudi Pharm. J.* 25 638–643. 10.1016/j.jsps.2017.04.037 28579904 PMC5447451

[B59] Xiao X. He Z. Cao W. Cai F. Zhang L. Huang Q. (2016). Oridonin inhibits gefitinib-resistant lung cancer cells by suppressing EGFR/ERK/MMP-12 and CIP2A/Akt signaling pathways. *Int. J. Oncol.* 48 2608–2618. 10.3892/ijo.2016.3488 27082429

[B60] Yao Z. Xie F. Li M. Liang Z. Xu W. Yang J. (2017). Oridonin induces autophagy via inhibition of glucose metabolism in p53-mutated colorectal cancer cells. *Cell Death Dis.* 8:e2633. 10.1038/cddis.2017.35 28230866 PMC5386482

[B61] Zacchino S. A. Butassi E. Cordisco E. Svetaz L. A. (2017a). Hybrid combinations containing natural products and antimicrobial drugs that interfere with bacterial and fungal biofilms. *Phytomedicine* 37 14–26. 10.1016/j.phymed.2017.10.021 29174600

[B62] Zacchino S. A. Butassi E. Di Liberto M. Raimondi M. Postigo A. Sortino M. (2017b). Plant phenolics and terpenoids as adjuvants of antibacterial and antifungal drugs. *Phytomedicine* 37 27–48. 10.1016/j.phymed.2017.10.018 29174958

[B63] Zhang C. Yang X. Zhang Q. Guo Q. He J. Qin Q. (2014). STAT3 inhibitor NSC74859 radiosensitizes esophageal cancer via the downregulation of HIF-1alpha. *Tumour Biol.* 35 9793–9799. 10.1007/s13277-014-2207-3 24981247

[B64] Zhou J. Hu Q. Zhang X. Zheng J. Xie B. Xu Z. (2018). Sensitivity to chemotherapeutics of NSCLC cells with acquired resistance to EGFR-TKIs is mediated by T790M mutation or epithelial-mesenchymal transition. *Oncol. Rep.* 39 1783–1792. 10.3892/or.2018.6242 29393480

[B65] Zuo G. Y. Wang C. J. Han J. Li Y. Q. Wang G. C. (2016). Synergism of coumarins from the Chinese drug *Zanthoxylum nitidum* with antibacterial agents against methicillin-resistant Staphylococcus aureus (MRSA). *Phytomedicine* 23 1814–1820. 10.1016/j.phymed.2016.11.001 27912884

